# Supplementary material for: Empirical methods for controlling false positives and estimating confidence in ChIP-Seq peaks
Source: BMC Bioinformatics. 2008 Dec 5;9:523. doi: 10.1186/1471-2105-9-523 (PMC2628906; doi:10.1186/1471-2105-9-523)
Supplement: Additional File 1 — A variety of html documents from the USeq web site detailing the available applications, their best usage, output file type descriptions, command line menus, etc. [file 1471-2105-9-523-S1.zip › USeqUserGuides/JavaDoc.zip!/JavaDoc/util/apps/HouseBean.html]

HouseBean


|  |  |  |
| --- | --- | --- |
| **Overview** \| **Package** \| **Class** \| **Use** \| **Tree** \| **Deprecated** \| **Index** \| **Help** | |  |
| **PREV CLASS**   **NEXT CLASS** | **FRAMES**    **NO FRAMES**     **All Classes** |  |
| SUMMARY: NESTED \| FIELD \| CONSTR \| METHOD | DETAIL: FIELD \| CONSTR \| METHOD |  |


---


## util.apps Class HouseBean

```
java.lang.Object
  util.apps.HouseBean
```

---

``` public class HouseBean extends java.lang.Object ```

---

**Constructor Summary**

`HouseBean(java.lang.String[] htmlLines)`


| **Method Summary** | |
| --- | --- |
| `double` | `getBathrooms()` |
| `int` | `getBedrooms()` |
| `java.lang.String` | `getCity()` |
| `int` | `getFamilyRooms()` |
| `int` | `getGarage()` |
| `double` | `getLatitude()` |
| `double` | `getLongitude()` |
| `int` | `getMls()` |
| `int` | `getPercentFinishedBasement()` |
| `int` | `getPrice()` |
| `int` | `getSize()` |
| `java.lang.String` | `getState()` |
| `java.lang.String` | `getStatus()` |
| `java.lang.String` | `getStreetAddress()` |
| `int` | `getYearBuilt()` |
| `int` | `getZipCode()` |
| `boolean` | `isBroken()` |
| `static void` | `main(java.lang.String[] args)` |
| `boolean` | `matchIt(java.util.regex.Pattern pat)` |
| `void` | `parseFields()` |
| `void` | `replaceQuotations()` |
| `void` | `setBathrooms(double bathrooms)` |
| `void` | `setBedrooms(int bedrooms)` |
| `void` | `setCity(java.lang.String city)` |
| `void` | `setFamilyRooms(int familyRooms)` |
| `void` | `setGarage(int garage)` |
| `void` | `setLatitude(double latitude)` |
| `void` | `setLongitude(double longitude)` |
| `void` | `setMls(int mls)` |
| `void` | `setPercentFinishedBasement(int percentFinishedBasement)` |
| `void` | `setPrice(int price)` |
| `void` | `setSize(int size)` |
| `void` | `setState(java.lang.String state)` |
| `void` | `setStatus(java.lang.String status)` |
| `void` | `setStreetAddress(java.lang.String streetAddress)` |
| `void` | `setYearBuilt(int yearBuilt)` |
| `void` | `setZipCode(int zipCode)` |
| `java.lang.String` | `toString()` |

**Methods inherited from class java.lang.Object**

`equals, getClass, hashCode, notify, notifyAll, wait, wait, wait`

**Constructor Detail**

### HouseBean

```
public HouseBean(java.lang.String[] htmlLines)
```


**Method Detail**

### toString

```
public java.lang.String toString()
```

:   **Overrides:**: `toString` in class `java.lang.Object`

---


### replaceQuotations

```
public void replaceQuotations()
```

---


### parseFields

```
public void parseFields()
```

---


### matchIt

```
public boolean matchIt(java.util.regex.Pattern pat)
```

---


### main

```
public static void main(java.lang.String[] args)
```

---


### getBathrooms

```
public double getBathrooms()
```

---


### setBathrooms

```
public void setBathrooms(double bathrooms)
```

---


### getBedrooms

```
public int getBedrooms()
```

---


### setBedrooms

```
public void setBedrooms(int bedrooms)
```

---


### getCity

```
public java.lang.String getCity()
```

---


### setCity

```
public void setCity(java.lang.String city)
```

---


### getFamilyRooms

```
public int getFamilyRooms()
```

---


### setFamilyRooms

```
public void setFamilyRooms(int familyRooms)
```

---


### getGarage

```
public int getGarage()
```

---


### setGarage

```
public void setGarage(int garage)
```

---


### getLatitude

```
public double getLatitude()
```

---


### setLatitude

```
public void setLatitude(double latitude)
```

---


### getLongitude

```
public double getLongitude()
```

---


### setLongitude

```
public void setLongitude(double longitude)
```

---


### getMls

```
public int getMls()
```

---


### setMls

```
public void setMls(int mls)
```

---


### getPercentFinishedBasement

```
public int getPercentFinishedBasement()
```

---


### setPercentFinishedBasement

```
public void setPercentFinishedBasement(int percentFinishedBasement)
```

---


### getPrice

```
public int getPrice()
```

---


### setPrice

```
public void setPrice(int price)
```

---


### getSize

```
public int getSize()
```

---


### setSize

```
public void setSize(int size)
```

---


### getState

```
public java.lang.String getState()
```

---


### setState

```
public void setState(java.lang.String state)
```

---


### getStatus

```
public java.lang.String getStatus()
```

---


### setStatus

```
public void setStatus(java.lang.String status)
```

---


### getStreetAddress

```
public java.lang.String getStreetAddress()
```

---


### setStreetAddress

```
public void setStreetAddress(java.lang.String streetAddress)
```

---


### getYearBuilt

```
public int getYearBuilt()
```

---


### setYearBuilt

```
public void setYearBuilt(int yearBuilt)
```

---


### getZipCode

```
public int getZipCode()
```

---


### setZipCode

```
public void setZipCode(int zipCode)
```

---


### isBroken

```
public boolean isBroken()
```


---


|  |  |  |
| --- | --- | --- |
| **Overview** \| **Package** \| **Class** \| **Use** \| **Tree** \| **Deprecated** \| **Index** \| **Help** | |  |
| **PREV CLASS**   **NEXT CLASS** | **FRAMES**    **NO FRAMES**     **All Classes** |  |
| SUMMARY: NESTED \| FIELD \| CONSTR \| METHOD | DETAIL: FIELD \| CONSTR \| METHOD |  |


---
